# Validation of the Khorana score in acute myeloid leukemia patients: a single-institution experience

**DOI:** 10.1186/s12959-019-0202-z

**Published:** 2019-07-02

**Authors:** Abu-Sayeef Mirza, Seongseok Yun, Najla Al Ali, Hannah Shin, Joseph Luke O’Neil, Maher Elharake, Daniel Schwartz, Katherine Robinson, Ethan Nowell, Grace Engle, Ibraahim Badat, Thomas Brimer, Amra Kuc, Ashton Sequeira, Sabbir Mirza, Dhiraj Sikaria, Jesus Diaz Vera, Noah Hackney, Sammy Abusrur, Jose Jesurajan, Jameson Kuang, Shreyans Patel, Sabrina Khalil, Sonya Bhaskar, Alexander Beard, Toaa Abuelenen, Kevin Ratnasamy, Nathan Visweshwar, Rami Komrokji, Michael Jaglal

**Affiliations:** 1 Department of Internal Medicine, H. Lee Moffitt Cancer Center and Research Institution, University of South Florida, 17 Davis Blvd., Suite 308, Tampa, FL 33606 USA; 2 Department of Malignant Hematology, H. Lee Moffitt Cancer Center and Research Institution, Tampa, FL USA; 3 Morsani College of Medicine, University of South Florida, Tampa, FL USA; 4 College of Arts and Sciences, University of South Florida, Tampa, FL USA

**Keywords:** Acute myeloid leukemia, Khorana score, Thrombosis, Risk prediction

## Abstract

**Background:**

Although patients with acute myeloid leukemia (AML) were shown to have an increased risk of thrombosis, no thrombosis risk assessment scoring system has been developed for AML patients. The Khorana Risk Score (KRS), which has been widely used for thrombosis risk assessment in the clinical setting, was developed on the basis of solid tumor data and has not been validated among AML patients. This study aims to validate the use of the KRS as a thrombosis risk-scoring system among patients with AML.

**Methods:**

Using data from H. Lee Moffitt Cancer Center and Research Institution’s Total Cancer Care Research Study, we retrospectively identified patients who were histologically confirmed with AML from 2000 to 2018. Clinical and laboratory variables at the time of AML diagnosis were characterized and analyzed. The thrombotic event rate was estimated with the Kaplan-Meier method and compared using the log-rank test.

**Results:**

A total of 867 AML patients were included in the analysis. The median age at AML diagnosis was 75 years (range, 51–96), and the majority were male (65%, *n* = 565). A total of 22% (*n* = 191), 51% (*n* = 445), 24% (*n* = 207), and 3% (*n* = 24) of patients had a KRS of 0, 1, 2, and 3, respectively. A total of 42 thrombotic events (3% [*n* = 6/191] with a KRS of 1; 5% [*n* = 23/445] with a KRS of 2; 6.3% [*n* = 13/207] with a KRS of 3) were observed, with a median follow-up of 3 months (range, 0.1–307). There was no statistical difference in the risk of thrombosis between these groups (*P* = .1949).

**Conclusions:**

Although there was an increased risk of thrombosis associated with a higher KRS among AML patients with a KRS of 1 to 3, the difference was not statistically significant. Furthermore, only a few patients were found to have a KRS > 3, and this was largely due to pancytopenia, which is commonly associated with AML. These results indicate the need for a better thrombotic risk-scoring system for AML patients.

## Background

Both solid and hematologic malignancies are associated with an increased risk of thromboembolic events [1]. One of the most commonly used risk-scoring systems for thrombosis, the Khorana Risk Score (KRS), was developed by studying patients with non-hematologic cancers receiving outpatient chemotherapy treatments. The KRS is based on body mass index (BMI) and white blood cell, hemoglobin, and platelet counts [2]. The KRS has proven to be the best validated risk assessment model for predicting thrombotic events among patients with solid tumors, given the hypercoagulable state of solid tumor malignancies [2, 3]. Despite its initial application in the ambulatory setting, its utility in inpatient settings also became evident [4].

However, the risk of developing thrombosis varies depending on the type of malignancy, and over the last decade, researchers have tried to identify the varying risks of thrombosis [3]. Recent research has shown that the KRS cannot be used to predict thrombosis among patients with certain solid organ malignancies [5, 6]. Among patients with lymphoid malignancies, such as diffuse large B cell lymphoma, the KRS was found to be inadequate for stratifying thrombosis risk, and other factors were found to be more predictive of thrombosis [7]. Therefore, a more specific prognostic scoring system was required to predict thrombosis in lymphomatous malignancies [8, 9].

Unlike patients with solid tumors, patients with acute leukemia commonly present with pancytopenia at the time of diagnosis [10]. Paradoxically, there is a risk of thrombosis in thrombocytopenic patients with hematological malignancies, but management with anticoagulation is often curtailed [11]. According to a 2017 meta-analysis, the incidence rate of thromboembolic events among patients with acute myeloid leukemia (AML) was approximately 6% [1]. According to current literature, the KRS has not been validated among patients with AML [12]. Furthermore, prophylactic and therapeutic management of thrombosis has not been standardized for patients with AML [13–15]. Thus, the goal of our current study was to validate the use of the KRS as a risk-scoring system for thrombosis among patients with AML.

## Methods and materials

### Patients and sample acquisition

Using data from H. Lee Moffitt Cancer Center and Research Institution’s Total Cancer Care Research Study, we retrospectively identified patients who were histologically confirmed with AML from 2000 to 2018. Patients with primary and secondary AML were included. Clinical and laboratory variables at the time of AML diagnosis (including age, sex, previous cancer history, use of growth factors, underlying coagulopathy, and white blood cell, hemoglobin, and platelet counts) were characterized and annotated using descriptive statistics. Information regarding venous thrombosis after AML diagnosis and prior thrombotic events before AML diagnosis were collected from individual patient chart reviews. Additional clinical information, including cytogenetics, AML risk stratification, treatment regimens, and Eastern Cooperative Oncology Group performance statuses, were also obtained. This study was approved by the H. Lee Moffitt Cancer Center and Research Institution Scientific Review Committee (MCC #18648) and the University of South Florida Institutional Review Board (**Pro00025683)**. Study data were collected and managed by using REDCap electronic data capture tools hosted at the University of South Florida [16].

### Primary end point and outcome measure

The primary end point of our study was the rate of venous thromboembolic event according to the KRS and arterial embolic events were excluded from our analysis. We included both superficial and deep vein thrombotic events as our end point. Thrombosis was counted as an event when VTE was confirmed by imaging modalities including venous duplex ultrasound and CT angiogram. Also, we counted VTE as an event when there is a clear documentation on the medical chart in patients who were transferred from another medical center.

### KRS calculation and statistical analyses

To calculate the KRS, we used complete blood count data from immediately before induction chemotherapy. Each of the following categories constituted a single point: pre-chemotherapy platelet count > 350 × 10^9^/L, leukocyte count > 11 × 10^9^/L, hemoglobin count < 10 g/dL, the use of erythropoiesis-stimulating agents, and a BMI > 35 kg/m^2^ [2]. Intermediate-risk patients had 1 to 2 points, and high-risk patients had > 3 points. A cancer type was designated as “other” with a score of zero indicating that it was not associated with thrombosis per KRS. The VTE rate was estimated with the Kaplan-Meier method, and univariate comparisons were completed using the log-rank test. A *P* value < .05 was regarded as statistically significant. We performed additional multivariate analysis utilizing clinical and laboratory parameters such as age, gender, race, BMI, prior history of cancer, and albumin. All statistical analyses were performed using SPSS v24.0 (SAS Institute Inc., Cary, NC, USA) and GraphPad Prism 7.00 (GraphPad Software, La Jolla, California, USA).

## Results

### Patient characteristics

A total of 867 AML patients were included in this study, of whom 44% (*n* = 383) and 56% (*n* = 483) had de novo and secondary AML, respectively. The median age at AML diagnosis was 75 years (range, 51–96), and the majority of patients were male (65%, *n* = 565). A total of 28% (*n* = 241) of patients had a prior history of cancer (hematologic malignancies, *n* = 34; solid tumors, *n* = 207). A total of 14% (*n* = 126), had a prior history of thrombosis, however information about whether it was venous or provoked was incomplete. A total of 12% (*n* = 101), 7% (*n* = 58), and 1% (*n* = 8) of patients were treated with erythropoietin-stimulating agents, granulocyte colony-stimulating factor, or granulocyte-macrophage colony-stimulating factor, respectively, before AML diagnosis. Eighty-four percent (*n* = 728) of patients were treated with growth factors of any type after AML diagnosis. The median BMI of patients was 27.1 (range, 14.8–103), and 5% (*n* = 47) of patients had a BMI > 35. A total of 26% (*n* = 229) of patients had a white blood cell count ≥ 11 × 10^9^/L; 67% (*n* = 584) of patients had a hemoglobin count < 10 g/L; and 1% (*n* = 10) of patients had a platelet count > 350 × 109/L. Additional demographic profiles and clinical parameters are described in Table 1.

**Table 1:** Additional clinical characteristics and demographic profiles of patients

Patient Characteristics	Value (*N* = 867)
Patient age	
median (range), y	75 (51–96)
Sex, No. (%)	
Male	565 (65)
Female	302 (35)
Race, No. (%)	
Caucasian	801 (92)
African American	18 (2)
Hispanic	25 (3)
Other	19 (2)
AML classification, No. (%)	
De novo AML	383 (44)
Secondary AML	483 (56)
AML risk stratification, No. (%)	
Favorable-risk group	21 (2)
Intermediate-risk group	485 (56)
Poor-risk group	266 (31)
History of prior or concurrent cancer, No. (%)	
Hematologic malignancies	34 (4)
Solid tumors	207 (24)
Prior thrombosis (arterial/venous)	126 (14)
Growth factors, No. (%)	
Before AML diagnosis	167 (19)
EPO	101 (12)
G-CSF	58 (7)
GM-CSF	8 (1)
Post AML diagnosis	728 (84)
Complete blood counts at diagnosis, No. (range)	
WBC (× 10^9^/L)	3.165 (0.08–413.74)
Hemoglobin (g/dL)	9.3 (5.6–15.2)
Platelet (× 10^9^/L)	46 (1–800)
Treatment regimen, No. (%)	
Chemotherapy	250 (29)
Hypomethylating agent	240 (28)
Best supportive care	225 (26)
BMI, No. (%)	
≤ 35 kg/m^2^	820 (65)
> 35 kg/m^2^	47 (5)

*Abbreviations*: *AML* acute myeloid leukemia, *BMI* body mass index, *EPO* erythropoietin, *G-CSF* granulocyte colony-stimulating factor, *GM-CSF* granulocyte-macrophage colony-stimulating factor, *WBC* white blood cells

### KRS and thrombosis risk analyses

**Fig. 1:**
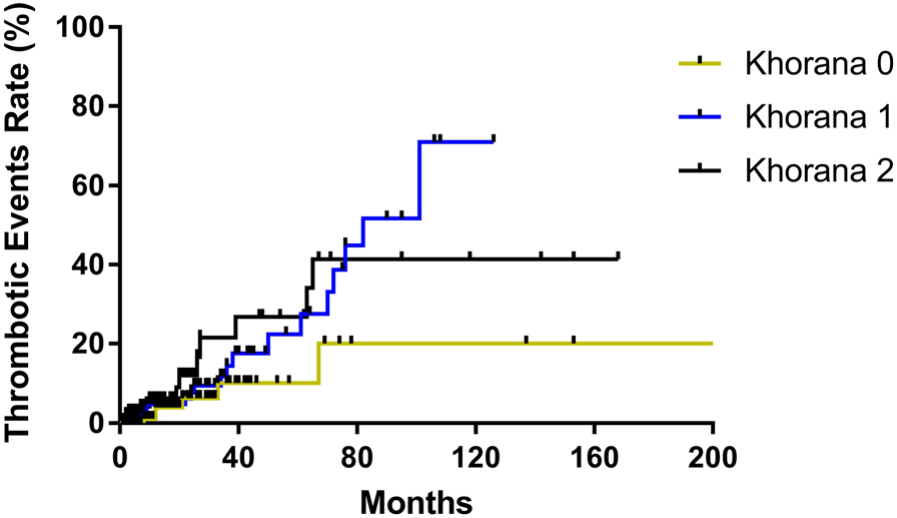
Risk of Thrombosis. VTE rates are plotted based on Khorana Score over time. AML patients with KRS 3 and 4 are not included in the analysis since there was no observed VTE in these groups

A total of 22% (*n* = 191), 51% (*n* = 445), 24% (*n* = 207), and 3% (*n* = 24) of patients had a KRS of 0, 1, 2, and 3, respectively **(**Table 2**)**. A total of 42 thrombotic events were observed, with a median follow-up of 3 months (range, 0.1–307). A total of 3% (*n* = 6/191), 5% (*n* = 23/445), 6.3% (*n* = 13/207), and 0% (*n* = 0/24) of these thrombotic events occurred among patients with a KRS of 0, 1, 2, and 3, respectively **(**Fig. 1**)**. A log-rank (Mantel-Cox) test showed no statistical difference of thrombosis risk between individual subgroups (*P* = 0.1949). In the comparison between patients with KRS 0 vs. KRS 1–3, there was no statistical difference in the rates of VTE between these two groups (3% vs. 5%, *P* = 0.2555) **(**Table 2**)**. In an additional multivariate analysis including age (HR 1.056, 95%CI 0.983–1.134, *P* = 0.135), gender (HR 1.118, 95%CI 0.466–2.678, *P* = 0.803), race (HR 0.998, 95%CI 0.977–1.021, *P* = 0.890), BMI (HR 1.030, 95%CI 0.984–1.079, *P* = 0.207), prior history of cancer (HR 0.422, 95%CI 0.129–1.387, *P* = 0.155), and albumin (HR 0.808, 95%CI 0.473–1.379, *P* = 0.434), there was no clinical or laboratory parameter that was significantly associated with increased risk of VTE.

**Table 2:** Khorana Risk Score and Thrombosis Event Correlation. Fisher’s Exact test showed that there is no statistical VTE difference between AML patients with KRS 0 vs. KRS 1–3 (*P* = 0.2555). Also, a log-rank (Mantel-Cox) test showed no statistical difference of VTE risk between individual subgroups (*P* = 0.1949)

Khorana Score	No. (%) of patients, *n* = 867	No. (%) of thrombosis events
0	191 (22)	6 (3)
1	445 (51)	23 (5)
2	207 (24)	13 (6.3)
3	24 (3)	0 (0)
1, 2, 3, and 4 (all patients)	867 (100)	42 (5)

## Discussion

Patients with AML were shown to have an increased risk of thrombosis, and thrombotic episodes represent significant morbidity and mortality among AML patients [17–19]. Although the KRS is the most widely used validated risk assessment model for predicting thrombotic events, it was not developed specifically for AML patients. Current predictive and prognostic models that are widely used in clinical settings do not take into account several risk factors that are relevant to AML patients [20, 21]. Therefore, we performed a single-institution retrospective study to validate the use of KRS in assessing the risk of thrombosis among AML patients.

We observed an increased number of VTE among AML patients with a KRS of 1 to 3 compared to patients with KRS 0. However, there was no statistical difference of thrombosis risk between these groups. Furthermore, only a few patients had a KRS ≥ 3, indicating that the current KRS system is not optimal to categorize the thrombosis risk group. This limitation of the KRS for AML patients is largely due to unique clinical characteristics associated with this patient group (such as pancytopenia at the time of diagnosis). Moreover, the KRS system does not include other clinical parameters—such as female sex, older age, number of chronic comorbidities, and presence of a central venous catheter—that were previously shown to be associated with an increased risk of thrombosis among AML patients [22]. Hematopoietic growth factors (erythropoietin, granulocyte colony-stimulating factor, and granulocyte-macrophage colony-stimulating factor) play a role in activating coagulation factors and cytokines that alter coagulation, modulate hemostasis, and cause platelet aggregation—all of which contribute to thrombotic events [23–27]. In addition, the KRS does not stratify by type of thrombosis, and arterial thromboembolism may be associated with greater morbidity and mortality than venous thromboembolism [27, 28].

The pathophysiology of arterial and venous thrombosis both follow the principles of Virchow’s Triad: alterations in flow, abnormal cell and molecular properties, and altered blood vessel walls [27]. The multifactorial pathogenesis of thrombosis in leukemia involves blast cells secreting prothrombotic tissue factor, cancer procoagulants, and cytokines. When combined with the release of growth factors, cytokines activate platelets [11]. These interactions are multiplied when chemotherapy induces massive cell death, which may explain why thrombosis can occur while leukemic patients are thrombocytopenic [22, 29]. According to one study, the majority (75%) of thrombotic events consist of deep vein thrombosis/pulmonary embolism, cardiac events, and cerebrovascular accidents, > 80% of which occur before or during induction chemotherapy [30]. High-risk patients who are taking chemotherapy have been a highlighted sub-population in recent studies who may benefit from routine thromboprophylaxis (https://www.nejm.org/doi/full/10.1056/NEJMoa1814630, https://www.nejm.org/doi/full/10.1056/NEJMoa1814468).

In acute promyelocytic leukemia, several factors were related to a higher incidence of thrombosis: leukocytes > 10 × 109/L (9% vs 4%, *P*  < .01), M3-variant subtype (11% vs 4%, *P* = .02), fibrinogen < 170 mg/dl (7% vs 3%, *P* = .02), and hemoglobin > 10 g/dl (8% vs 4%, *P* = .03) [31]. In addition to previously known prognostic factors, independent risk factors for thrombosis also include increasing age and cytogenetic risk [32, 33]. Recently, disseminated intravascular coagulation was found to be significantly predictive of venous and arterial thrombosis, with thrombosis incidence being approximately 10% among patients who were treated with multiple rounds of chemotherapy [34]. Recent data suggest that malignant leukocytes mediate both disseminated intravascular coagulation and primary hyperfibrinolysis in acute promyelocytic leukemia [35]. Additionally, both hyperleukocytosis and platelet aggregation seem to contribute to arterial thrombosis in acute leukemia [36, 37]. Collectively, these results suggest that further studies are warranted to develop an optimal thrombosis risk-scoring system that reflects the unique clinical and pathogenic features of AML patients.

Although limited by its retrospective nature, this study involved the experience of a single institution from 2000 to 2018, during which time the institution used the same diagnostic and treatment protocols and maintained follow-up data in a large comprehensive database. A large group of trained personnel conducted the extensive chart review but any threats to data quality was mitigated by using a centralized, organized extraction tool via REDCap. Another limitation of this study was the lack of routine screening for thrombotic events. Only symptomatic events were included, which may have falsely decreased observed frequency rates.

## Conclusions

Among AML patients with a KRS of 1 to 3, we found a higher incidence of thrombotic events; however, the difference between these groups was not statistically significant. The proportion of patients with a KRS ≥ 3 was relatively low, which is largely due to pancytopenia, a common presentation among AML patients. These results suggest that the development of a better thrombotic risk-scoring system is warranted for AML patients.

## Data Availability

All data and materials can be made available upon reasonable request.

## Abbreviations

AML: Acute Myeloid Leukemia
BMI: Body Mass Index
KRS: Khorana Risk Score

## References

[1] Wu YY, Tang L, Wang MH (2017). Leukemia and risk of venous thromboembolism: a meta-analysis and systematic review of 144 studies comprising 162,126 patients. Sci Rep.

[2] Khorana AA, Kuderer NM, Culakova E, Lyman GH, Francis CW (2008). Development and validation of a predictive model for chemotherapy-associated thrombosis. Blood.

[3] Khorana AA, Francis CW (2018). Risk prediction of cancer-associated thrombosis: appraising the first decade and developing the future. Thromb Res.

[4] Parker A, Peterson E, Lee AYY, de Wit C, Carrier M, Polley G, Tien J, Wu C (2018). Risk stratification for the development of venous thromboembolism in hospitalized patients with cancer. J Thromb Haemost.

[5] van Es N, Franke VF, Middeldorp S, Wilmink JW, Buller HR (2017). The Khorana score for the prediction of venous thromboembolism in patients with pancreatic cancer. Thromb Res.

[6] Wang Y, Attar BM, Fuentes HE, Yu J, Zhang H, Tafur AJ (2018). Performance of Khorana risk score for prediction of venous thromboembolism in patients with hepatocellular carcinoma. Clin Appl Thromb Hemost.

[7] Rupa-Matysek J, Gil L, Kazmierczak M, Baranska M, Komarnicki M (2017). Prediction of venous thromboembolism in newly diagnosed patients treated for lymphoid malignancies: validation of the Khorana risk score. Med Oncol.

[8] Antic D, Biljana M, Milic N, Cheson BD, Narkhede M, Panovska I, Trajkova S, Popova M, Llamas P, Garcías Raso A (2017). Development and external validation of thrombosis lymphoma /Throly/ score–interim analysis. Blood.

[9] Antic D, Biljana M, Milic N, Cheson BD, Narkhede M, Panovska I, Trajkova S, Popova M, Llamas P, Raso AG (2018). Internal and external validation of THROLY (thrombosis lymphoma) score. Thromb Res.

[10] Swords R, Santini V (2012). In elderly patients with AML, which patients should be considered fit or unfit for standard induction therapy?. Hematology Am Soc Hematol Educ Program.

[11] Del Principe MI, Del Principe D, Venditti A (2017). Thrombosis in adult patients with acute leukemia. Curr Opin Oncol.

[12] Sud R, Khorana AA (2009). Cancer-associated thrombosis: risk factors, candidate biomarkers and a risk model. Thromb Res.

[13] Zimran E, Hoffman R, Kremyanskaya M (2018). Current approaches to challenging scenarios in myeloproliferative neoplasms. Expert Rev Anticancer Ther.

[14] Khan M, Cox TM, Nassif M, Alzubaidi MA, Garg N, Qiao W, Aung FM, Oo TH, Rojas-Hernandez CM (2018). Comparative outcomes of thrombocytopenic acute leukemic patients with venous thromboembolism at a Comprehensive Cancer Center. J Thromb Thrombolysis.

[15] Farge D, Debourdeau P, Beckers M, Baglin C, Bauersachs RM, Brenner B, Brilhante D, Falanga A, Gerotzafias GT, Haim N (2013). International clinical practice guidelines for the treatment and prophylaxis of venous thromboembolism in patients with cancer. J Thromb Haemost.

[16] Harris PA, Taylor R, Thielke R, Payne J, Gonzalez N, Conde JG (2009). Research electronic data capture (REDCap)—a metadata-driven methodology and workflow process for providing translational research informatics support. J Biomed Inform.

[17] Stone RM (2016). Thrombosis in AML? Yes, but when to worry?. Blood.

[18] Streiff MB, McCrae KR, Kuderer NM, Milentijevic D, Germain G, Laliberté F, Le N, Lefebvre P, Lyman GH, Khorana AA (2018). Healthcare costs in patients with Cancer increase with increasing risk of venous thromboembolism. Blood.

[19] Shrotriya S, Dhakal P, Sharma M, Gardiner J, Al-Janadi A, Lupi A, Delshad A, Rayamajhi S (2018). CAN WE predict risk factors of venous thromboembolism among Cancer inpatients?. Blood.

[20] Al-Ani F, Wang YP, Lazo-Langner A (2018). Development of a clinical prediction rule for the risk of venous thromboembolism in patients with acute leukemia. Blood.

[21] Khorana AA, Francis CW, Culakova E, Kuderer NM, Lyman GH (2007). Thromboembolism is a leading cause of death in cancer patients receiving outpatient chemotherapy. J Thromb Haemost.

[22] Vu K, Luong NV, Hubbard J, Zalpour A, Faderl S, Thomas DA, Yang D, Kantarjian H, Kroll MH (2015). A retrospective study of venous thromboembolism in acute leukemia patients treated at the University of Texas MD Anderson Cancer Center. Cancer Med.

[23] Bussolino F, Ziche M, Wang JM, Alessi D, Morbidelli L, Cremona O, Bosia A, Marchisio PC, Mantovani A (1991). In vitro and in vivo activation of endothelial cells by colony-stimulating factors. J Clin Invest.

[24] Kawachi Y, Watanabe A, Uchida T, Yoshizawa K, Kurooka N, Setsu K (1996). Acute arterial thrombosis due to platelet aggregation in a patient receiving granulocyte colony-stimulating factor. Br J Haematol.

[25] Nadir Y, Hoffman R, Brenner B (2004). Drug-related thrombosis in hematologic malignancies. Rev Clin Exp Hematol.

[26] Sisson SD, Dinarello CA (1988). Production of interleukin-1 alpha, interleukin-1 beta and tumor necrosis factor by human mononuclear cells stimulated with granulocyte-macrophage colony-stimulating factor. Blood.

[27] Visweshwar N, Jaglal M, Sokol L, et al. Hematological malignancies and arterial thromboembolism. Indian J Hematol Blood Transfus. 2019;1–14. 10.1007/s12288-019-01085-x, https://link.springer.com/article/10.1007%2Fs12288-019-01085-x. 10.1007/s12288-019-01085-x PMC6825093 31741612

[28] Khorana AA, Francis CW, Culakova E, Fisher RI, Kuderer NM, Lyman GH (2006). Thromboembolism in hospitalized neutropenic cancer patients. J Clin Oncol.

[29] Lebois M, Josefsson EC (2016). Regulation of platelet lifespan by apoptosis. Platelets.

[30] Rashidi A, Silverberg ML, Conkling PR, Fisher SI (2013). Thrombosis in acute promyelocytic leukemia. Thromb Res.

[31] Montesinos P, de la Serna J, Vellenga E, Rayon C, Bergua J, Parody R, Esteve J, Gonzalez M, Brunet S, Sanz M (2006). Incidence and risk factors for thrombosis in patients with acute Promyelocytic leukemia. Experience of the PETHEMA LPA96 and LPA99 protocols. Blood.

[32] Lee YG, Kim I, Kwon JH, Yoon SS, Park S, Song L, Yoon JH, Shin SH, Min WS, Kim HJ (2015). Implications of cytogenetics for venous thromboembolism in acute myeloid leukaemia. Thromb Haemost.

[33] O'Donnell MR, Tallman MS, Abboud CN, Altman JK, Appelbaum FR, Arber DA, Bhatt V, Bixby D, Blum W, Coutre SE (2017). Acute Myeloid Leukemia, Version 3.2017, NCCN clinical practice guidelines in oncology. J Natl Compr Cancer Netw.

[34] Libourel EJ, Klerk CPW, van Norden Y, de Maat MPM, Kruip MJ, Sonneveld P, Löwenberg B, Leebeek FWG (2016). Disseminated intravascular coagulation at diagnosis is a strong predictor for thrombosis in acute myeloid leukemia. Blood.

[35] Mantha S, Tallman MS, Soff GA (2016). What’s new in the pathogenesis of the coagulopathy in acute promyelocytic leukemia?. Curr Opin Hematol.

[36] Fass R, Haddad M, Zaizov R, Sandbank Y, Yaniv I, Cohen IJ, Stark B, Zelikovski A (1992). Recurrent peripheral arterial occlusion by leukemic cells sedimentation in acute promyelocytic leukemia. J Pediatr Surg.

[37] Kafetzakis A, Foundoulakis A, Ioannou CV, Stavroulaki E, Koutsopoulos A, Katsamouris AN (2007). Acute lower limb ischemia as the initial symptom of acute myeloid leukemia. Vasc Med.

